# Identification of Key Genes Associated with Feed Utilization Efficiency in *Penaeus vannamei* Fed a Plant-Based Diet Using WGCNA

**DOI:** 10.3390/ani16101480

**Published:** 2026-05-12

**Authors:** Hao Zhang, Yuhao Xu, Juan Sui, Qiang Fu, Mianyu Liu, Jian Tan, Jie Kong, Kun Luo, Xianhong Meng, Sheng Luan, Ping Dai

**Affiliations:** 1 College of Fisheries and Life Science, Shanghai Ocean University, Shanghai 201306, China; zh18330251072@163.com (H.Z.); xuyh9391@126.com (Y.X.); 2 State Key Laboratory of Mariculture Biobreeding and Sustainable Goods, Yellow Sea Fisheries Research Institute, Chinese Academy of Fishery Science, Qingdao 266071, China; suijuan@ysfri.ac.cn (J.S.); fuqiang@ysfri.ac.cn (Q.F.); tanjian@ysfri.ac.cn (J.T.); kongjie@ysfri.ac.cn (J.K.); luokun@ysfri.ac.cn (K.L.); mengxianhong@ysfri.ac.cn (X.M.); 3 Wuxi College of Fisheries, Nanjing Agricultural University, Wuxi 214081, China; 2022213007@stu.njau.edu.cn

**Keywords:** *Penaeus vannamei*, plant-based diet, residual feed intake, WGCNA, hub genes

## Abstract

Overcoming fishmeal dependence is crucial for the sustainable development of *Penaeus vannamei* aquaculture, yet the molecular mechanisms underlying the efficient utilization of plant-based diets remain poorly understood. In this study, residual feed intake (RFI) was used as an indicator to investigate these mechanisms. Transcriptome sequencing of the intestine, hepatopancreas, and muscle was performed on shrimp fed a plant-based diet, followed by weighted gene co-expression network analysis (WGCNA) and validation in an independent population. Phenotypic analysis revealed extensive variation in growth performance and RFI among individuals fed the plant-based diet. WGCNA identified four tissue-specific modules significantly associated with RFI, from which 20 hub genes were screened. Independent validation confirmed that seven genes were significantly differentially expressed between high- and low-feed-efficiency groups (*q* < 0.1): five in the intestine (*LOC113811628*, *LOC113811631*, *LOC113824170*, *LOC113817752*, *LOC113811632*) and two in the hepatopancreas (*LOC113809216*, *LOC113820990*). These genes are involved in mitochondrial function, energy metabolism, intracellular pH homeostasis, and nucleic acid metabolism, suggesting potential coordination between the intestine and hepatopancreas in response to plant-based diets. Collectively, these findings provide insights into the genetic basis of shrimp adaptation to plant-based diets and offer candidate molecular targets that may inform selective breeding efforts aimed at improving feed efficiency in *P. vannamei*.

## Simple Summary

Replacing fishmeal with plant-based ingredients is essential for sustainable shrimp farming, but the molecular mechanisms enabling shrimp to efficiently utilize plant-based diets remain unclear. In this study, we investigated plant-based feed utilization efficiency in *Penaeus vannamei* using residual feed intake (RFI) as a key indicator. Through transcriptome sequencing and weighted gene co-expression network analysis (WGCNA), we identified seven genes associated with RFI that function in energy metabolism, nutrient digestion, and cellular homeostasis. These findings provide candidate genetic targets that may support selective breeding programs aimed at improving the utilization of plant-based feeds in shrimp aquaculture.

## 1. Introduction

The Pacific white shrimp (*Penaeus vannamei*) is widely cultivated around the world due to its rapid growth, strong resistance to disease and environmental stress, and broad salinity tolerance [1]. The continued expansion of this industry depends heavily on the development of more cost-effective feeds, as feed accounts for 40–60% of total production costs [2]. Protein is the core nutritional component supporting shrimp growth and development, and both its source and utilization efficiency directly influence economic returns and environmental sustainability in aquaculture. Fishmeal has long served as the primary protein source in shrimp feeds due to its comprehensive nutrient profile and high palatability, with inclusion levels in commercial diets typically exceeding 20% [3]. However, increasing pressure on global fishmeal supplies and sustained price increases have made the development of fishmeal replacement technologies an urgent priority for the sustainable development of the aquaculture industry [4,5]. Plant protein sources, such as soybean meal and peanut meal, are considered ideal alternatives to fishmeal due to their low cost and stable supply, and have already been incorporated into commercial feeds [6].

Although previous studies have demonstrated that reducing fishmeal inclusion to 5% in *P. vannamei* diets does not significantly impair growth performance, completely replacing fishmeal with plant protein sources remains challenges [1]. Using multiple families of *P. vannamei*, Dai et al. [7]. reported that when shrimp were fed a plant-based (fishmeal-free) diet, they exhibited significantly higher residual feed intake (RFI) and lower average daily weight gain (ADG) compared to those fed a fishmeal-based diet, suggesting that plant-based diets may reduce yields. Moreover, genetic correlations between diets for RFI and ADG were moderate to low (0.646 and 0.296, respectively), indicating pronounced genotype-by-diet interactions. These findings reveal substantial family-level variation in response to plant-based diet. Exploiting such family-level variation through genetic improvement therefore holds promise for developing shrimp strains with enhanced utilization efficiency of plant-based diets, thereby reducing production costs and promoting aquaculture sustainability.

To this end, unraveling the systemic adaptive mechanisms of *P. vannamei* under nutritional stress induced by plant-based diets has become a key research focus. Previous studies have explored shrimp responses to plant protein diets at physiological and molecular levels. For instance, replacing fishmeal with different soybean meal sources (fermented soybean meal and conventional soybean meal at 8% inclusion rates) has been shown to affect growth performance, enzyme activities, and the expression of key immune-related genes in *P. vannamei*, with stress responses induced by antigenic proteins identified as an important factor limiting feed utilization efficiency [8]. Similarly, replacing 50% of soybean meal with fermented rapeseed meal resulted in significantly increased residual feed and feed loss in *P. vannamei*, and transcriptomic analyses revealed corresponding changes in related signaling pathways [9]. However, the systemic, multi-tissue molecular regulatory mechanisms underlying the response of *P. vannamei* to nutritional stress caused by plant-based diets remain largely unresolved. Feed utilization efficiency represents the integrated outcome of a series of complex physiological processes, including digestion, absorption, metabolism, and energy allocation, and can serve as an ideal link between macroscopic phenotypic responses to nutritional stress and their underlying molecular mechanisms [10].

Weighted gene co-expression network analysis (WGCNA) is a powerful systems biology approach that enables the identification of gene modules exhibiting highly coordinated expression patterns associated with target traits from transcriptomic data, making it particularly well suited for elucidating the molecular mechanisms underlying complex traits [11,12]. This approach has been successfully applied to investigate stress-related biological responses in various aquatic species, highlighting its utility in identifying key pathways and core regulatory genes. For example, WGCNA has been used to reveal strong negative correlations between specific metabolites within co-expression modules and the gut microbiota in *P. vannamei* under pesticide exposure, demonstrating its potential for linking metabolic responses with microbial community changes [13]. In addition, WGCNA-based analyses have systematically characterized gene expression dynamics in the gills and hepatopancreas of *Macrobrachium rosenbergii* under hypoxia–reoxygenation stress, identifying key modules and candidate genes involved in stress adaptation and metabolic regulation, including those associated with the HIF-1 signaling pathway [14]. Similarly, WGCNA has been applied to investigate thermal tolerance in the Manila clam (*Ruditapes philippinarum*), identifying multiple gene modules significantly associated with temperature and revealing key molecular mechanisms related to cellular metabolism, apoptosis, and endoplasmic reticulum stress [15]. Despite the broad applicability of WGCNA in stress-related studies, to date, no studies have employed this approach to systematically analyze feed utilization efficiency in *P. vannamei* fed plant-based diets.

In this study, *P. vannamei* were reared under a plant-based diet to systematically evaluate their feed utilization efficiency. Multi-tissue transcriptome sequencing was performed on key metabolic and digestive organs, including the hepatopancreas, intestine, and muscle. By applying multi-tissue WGCNA, we identified co-expression modules significantly associated with feed utilization efficiency and further screened key candidate genes potentially regulating the utilization efficiency of plant-based diets. Collectively, this study aims to systematically elucidate the molecular genetic basis underlying the adaptation of *P. vannamei* to plant-based diets, thereby providing a theoretical foundation for the genetic breeding of shrimp strains with enhanced utilization efficiency of plant-based diets.

## 2. Materials and Methods

### 2.1. Experimental Design

In this study, a plant-based (fishmeal-free) diet was formulated using soybean meal and peanut meal as fishmeal replacements (0% fishmeal, 38% crude protein) for the feeding experiment. The experimental pelleted diets were prepared following the methods described by Xu et al. [16]. The ingredients and proximate composition of the experimental diet are presented in Table 1. Experimental *P. vannamei* were obtained from a commercial fast-growing strain. After being purchased at the post-larval (PL5) stage, shrimp were reared in circular culture tanks with a water volume of 2 m^3^. When the shrimp reached a body length of 6–8 cm, a total of 480 individuals were selected for the feeding experiment. The experiment was conducted using a recirculating aquaculture system equipped with individual culture units (20 cm × 15 cm × 10 cm, length × width × height). Each shrimp was housed individually in a separate culture unit. Water flowed through the entire system, but each unit was physically isolated from the others by solid side walls, so no direct contact between adjacent units occurred except for water exchange. A specially designed mesh installed at the bottom of each unit allowed feces to pass through while retaining feed particles. The experiment was carried out at the Whiteleg Shrimp Breeding Center of BLUP Aquabreed Co., Ltd., located in Weifang, Shandong Province, China.

### 2.2. Feeding Trial and Data Collection

Before the formal experiment, all shrimp underwent a one-week acclimation period during which they were fed a commercial fishmeal-based feed. During the trial, a separate plastic tube was prepared for each shrimp to hold its pelleted feeds. Shrimp were fed three times daily at 09:00, 14:00, and 18:00. The amount of diet per meal was carefully controlled to ensure consumption within half an hour. Any uneaten pellets were collected in a separate container half an hour after each feeding and then dried until a stable weight was achieved. Approximately 80% of the seawater in the tanks was exchanged daily, and the temperature was maintained at 27 ± 1 °C (mean ± SD), with salinity kept at approximately 30. In addition, dissolved oxygen was maintained at 6.0–7.0 mg/L through continuous aeration, and the photoperiod was set to follow the natural photoperiod (lights on from dawn to dusk). Molts and dead shrimp were removed daily.

The feeding trial was conducted from 27 September 2024 to 7 November 2024 and lasted for 42 days. One day before the experiment, the initial body weight ($W_{1}$) and initial feed weight ($F_{1}$) of each shrimp were recorded. At the end of the experiment, the harvest body weight ($W_{2}$), remaining feed weight ($F_{r}$), and total uneaten feed weight ($F_{0}$) of surviving shrimp were measured. Because sex could only be determined for a subset of individuals at the end of the experiment, sex information was not collected. ADG and daily feed intake (DFI) were calculated as follows:

$$\mathrm{ADG}\;=\;(W_{2}\;-W_{1})/42$$

$$\mathrm{DFI}=(F_{1}-F_{r}-F_{0})/42$$

RFI was used as the indicator of feed utilization efficiency. RFI was defined as the difference between the actual amount of feed intake and the expected feed intake. As energy intake from feed during the shrimp growth stage is primarily allocated to growth and maintenance, expected DFI was estimated using a nonlinear approximation model as follows:

$$DFI=b_{1}\times {MW}^{b_{2}}+b_{3}\times ADG$$

where MW is the mid-weight ($MW=W_{1}/2$ + $W_{2}/2$), ${MW}^{b_{2}}$ is the metabolic mid-weight, DFI and ADG are as described above, and b_1_, b_2_ and b_3_ are partial regression coefficients. With observed DFI serving as the dependent variable, the coefficients were derived using the nonlinear regression procedure nls implemented in R (version 4.2.3).

RFI was calculated as observed DFI minus expected DFI. A negative RFI means an animal consumes less feed than expected, while a positive RFI indicates higher feed intake. Animals with low RFI are more efficient, and those with high RFI are less efficient.

### 2.3. Transcriptome Sequencing

After the feeding trial, 50 individuals exhibiting clear differences in RFI were selected based on the RFI distribution of all surviving shrimp. For each individual, intestine, hepatopancreas, and muscle tissues were collected. Total RNA was extracted from all samples using TRIzol reagent (Invitrogen, Carlsbad, CA, USA). RNA purity and concentration were evaluated using a NanoDrop 2000 spectrophotometer (Thermo Scientific, Waltham, MA, USA), and RNA integrity was assessed using an Agilent 2100 Bioanalyzer (Agilent Technologies, Santa Clara, CA, USA).

For RNA samples that passed quality control, mRNA was enriched using Oligo (dT) magnetic beads to ensure high-quality mRNA. The enriched mRNA was fragmented for subsequent cDNA synthesis. First-strand cDNA was synthesized using random hexamer primers, followed by second-strand cDNA synthesis. cDNA libraries were constructed via adapter ligation and PCR amplification, and library quality was assessed using an Agilent 2100 Bioanalyzer. Qualified libraries were sequenced on an Illumina NovaSeq 6000 platform (Illumina, San Diego, CA, USA) to generate 150 bp paired-end reads. Library preparation and sequencing were conducted by Qingdao OE Biotech Co., Ltd., Qingdao, China.

Raw reads were subjected to quality control using fastp (version 0.23.4) [17] to obtain clean reads. Following genome index construction, clean reads were aligned to the reference genome (GCF_003789085.1, NCBI) [18] using HISAT2 (version 2.2.1) [19] to obtain genomic alignment information and sample-specific sequence features. Read counts mapped to protein-coding genes were obtained using HTSeq-count (version 2.0.1) [20], generating a raw expression matrix. Gene expression levels were calculated using the transcripts per million (TPM) method to normalize for gene length and sequencing depth, resulting in gene expression profiles suitable for subsequent analyses.

### 2.4. Construction of Weighted Gene Co-Expression Networks

Genes with low expression or low variability were filtered out based on the following criteria: mean TPM > 3, detection rate (proportion of samples with TPM > 0) > 60%, and coefficient of variation > 0.2, and the retained genes were used for WGCNA. Weighted gene co-expression networks were constructed using the WGCNA package (version 1.73) in R. Hierarchical clustering of samples was performed using the hclust function to assess sample relationships and identify potential outliers. To construct co-expression networks that satisfied scale-free topology criteria, the pickSoftThreshold function was used to determine the optimal soft-thresholding power, ensuring that the scale-free topology fit index (R^2^) reached a stable level while maintaining reasonable mean connectivity. Different soft-thresholding powers were applied for different tissues to ensure that each network exhibited appropriate scale-free topology characteristics. Based on the selected thresholds, the blockwiseModules function was used to construct co-expression networks and identify gene modules via dynamic tree cutting. The minimum module size was set to 50 genes. The first principal component of each module was calculated as the module eigengene (ME), representing the overall expression pattern of genes within that module. Modules with similar expression patterns were merged using a module merging threshold of 0.15. Within each tissue, every module was assigned a distinct color.

### 2.5. Identification of Trait-Specific Modules and Hub Genes

Pearson correlation coefficients (r) and corresponding *p*-values between module eigengenes and RFI were then calculated. Modules with an absolute correlation coefficient greater than 0.4 were defined as RFI-associated trait-specific modules. Gene connectivity within each module was calculated using the WGCNA package in R. Using the topological overlap matrix (TOM) obtained from the network construction step, the intra-module connectivity of a gene was defined as follows: first, a soft-thresholding power transformation was applied to the correlation coefficients between genes; then, for a given gene, the sum of the weighted edge strengths between it and all other genes within the same module was calculated. This metric reflects the centrality of that gene in the co-expression network. Generally, larger modules contain more genes, and the average connectivity per gene tends to be higher due to the greater number of neighbors. Since connectivity is a sum-based metric, genes in larger modules naturally tend to have higher absolute connectivity values. Functional annotation of module genes was performed based on the NCBI database. In each trait-specific module, the top five genes with functional annotations and the highest connectivity values were selected as hub genes.

### 2.6. Validation of Hub Genes

To validate the association between hub genes and RFI under a plant-based diet, an independent feeding experiment was conducted. In 2025, 30 newly established families were selected, and 15 individuals (6–8 cm body length) from each family were used, resulting in a total of 450 shrimp for RFI evaluation. The experiment was conducted from 17 July 2025 to 27 August 2025, lasting 42 days. Feeding management and trait measurement procedures were consistent with those described in Section 2.2. After the experiment, RFI phenotypes were calculated for all surviving shrimp. Based on the RFI distribution, 125 individuals with pronounced phenotypic differences were selected as the validation population. These individuals were ranked according to RFI values, and the 30 shrimp with the highest RFI values and the 30 shrimp with the lowest RFI values were defined as the low feed efficiency group and the high feed efficiency group, respectively. Intestine, hepatopancreas, and muscle tissues were collected from each individual, resulting in 30 samples per tissue type. All tissue samples were processed following the standardized procedures described in Section 2.3 for total RNA extraction, quality assessment, library construction, transcriptome sequencing, and TPM-based expression quantification.

For each hub gene, the normality of expression values within the high and low RFI groups were assessed using the Shapiro–Wilk test, and homogeneity of variances between the two groups were assessed using Levene’s test. If both assumptions were met for a given gene, an independent-sample *t*-test was applied; otherwise, the Mann–Whitney U test was used. Log_2_-transformed fold changes (log_2_FCs) were calculated for the low feed efficiency group relative to the high feed efficiency group. To control for multiple hypothesis testing, *p*-values were adjusted using the false discovery rate (FDR) method. To retain a broader set of candidate genes for subsequent molecular studies, adjusted *p*-values (*q*) < 0.1 were considered statistically significant. For the genes that were validated as significant, Pearson correlation analysis between their expression levels and ADG was conducted using all 60 individuals from both the high and low RFI groups, with *p* < 0.05 considered statistically significant.

## 3. Results

### 3.1. Descriptive Statistics of Phenotypes

**Figure 1:**
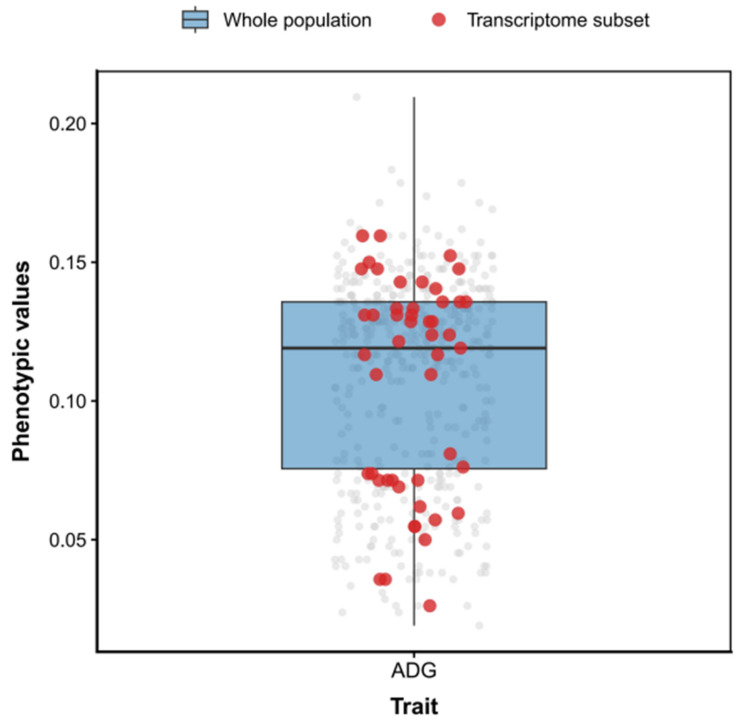
Boxplot of ADG phenotypes in *P. vannamei* under a plant-based diet. Note: Gray dots represent all individuals (*n* = 468); Red dots indicate the subset of individuals selected for transcriptome sequencing (*n* = 50).

**Figure 2:**
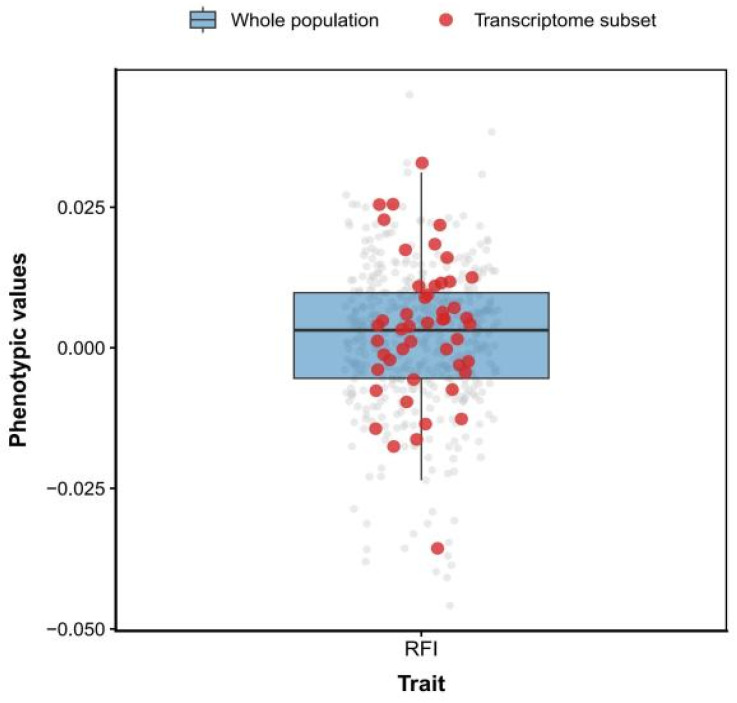
Boxplot of RFI phenotypes in *P. vannamei* under a plant-based diet. Note: Gray dots represent all individuals (*n* = 468); Red dots indicate the subset of individuals selected for transcriptome sequencing (*n* = 50).

At the end of the experiment, a total of 468 individuals were harvested. ADG values for the entire population ranged from 0.019 to 0.210 g/d (Figure 1), with a mean of 0.109 g/d, while RFI ranged from −0.046 to 0.045 g/d (Figure 2), with a mean of 0.002 g/d. In the subset of 50 individuals selected for transcriptome sequencing, ADG ranged from 0.026 to 0.160 g/d (Figure 1), with an average of 0.105 g/d, and RFI ranged from −0.036 to 0.033 g/d (Figure 2), with an average of 0.003 g/d. The selected 50 individuals largely cover the main variation range of the whole population in both ADG and RFI phenotypes, making them suitable for subsequent WGCNA.

### 3.2. Transcriptome Sequencing Statistics Across Tissues

Transcriptome sequencing of intestinal tissue from 50 individuals yielded 328.47 Gb of clean data, with per-individual data ranging from 5.87 to 7.06 Gb. The proportion of Q30 bases ranged from 95.79% to 97.58%, with an average GC content of 46.55%. Genome alignment rates ranged from 85.24% to 89.15%, resulting in the detection of 21,010 genes. For hepatopancreas tissue, sequencing generated 328.80 Gb of clean data, with per-individual data ranging from 5.93 to 7.06 Gb. Q30 bases ranged from 97.35% to 98.16%, with an average GC content of 48.74%. Genome alignment rates ranged from 85.97% to 89.47%, yielding 20,874 genes. For muscle tissue, sequencing produced 332.14 Gb of clean data, with per-individual data ranging from 5.91 to 7.06 Gb. Q30 bases ranged from 96.57% to 98.50%, with an average GC content of 52.30%. Genome alignment rates ranged from 76.93% to 92.72%, resulting in the identification of 19,188 genes.

### 3.3. Co-Expression Network Construction and Module Detection

**Figure 3:**
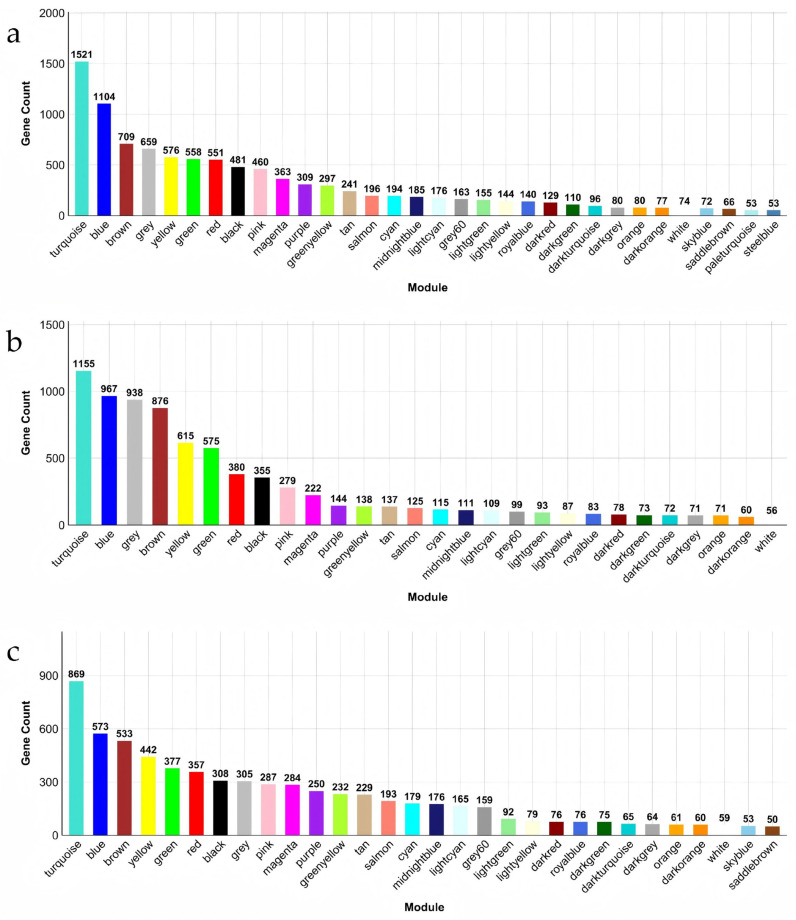
Distribution of co-expression modules in intestine (**a**), hepatopancreas (**b**) and muscle (**c**). Note: Bar plots illustrating the size distribution of co-expression modules (excluding the gray module) in intestine (**a**), hepatopancreas (**b**), and muscle (**c**). The *x*-axis represents module names (color-coded), and the *y*-axis represents the number of genes within each module. The fill color of each bar corresponds to the respective module color, providing a direct visual representation of module sizes. Modules are sorted in descending order of gene count. Numbers above each bar indicate the exact gene count for that module.

Prior to WGCNA, gene filtering was performed for each tissue. After quality control, 10,072 genes in the intestine, 8084 genes in the hepatopancreas, and 6728 genes in the muscle were retained. For the intestine, a soft-thresholding power of 5 was applied, resulting in 32 co-expression modules (Figure 3a). The turquoise module contained the largest number of genes (1521), whereas the steel blue module contained the fewest (53), with 659 genes remaining unassigned (gray module). For the hepatopancreas, a soft-thresholding power of 6 yielded 28 modules (Figure 3b), with the turquoise module containing the most genes (1155) and the white module the fewest (56), with 938 genes remaining unassigned. For the muscle, a soft-threshold power of 5 produced 30 modules (Figure 3c), with the turquoise module containing 869 genes and the saddle brown module the fewest (50), and 305 genes remaining unassigned.

### 3.4. Identification of Trait-Specific Modules

**Figure 4:**
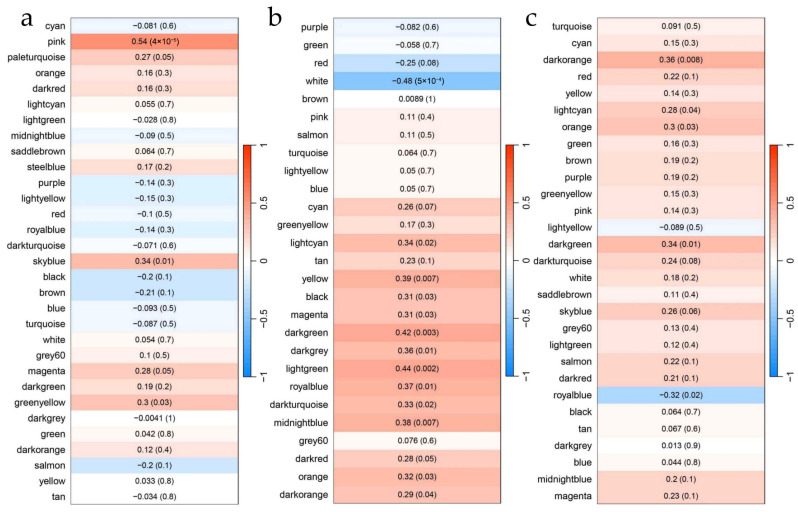
Module-trait correlation heatmaps of intestine (**a**), hepatopancreas (**b**) and muscle (**c**). Note: Heatmaps showing the correlation between module eigengenes and RFI. Each cell represents the Pearson correlation coefficient (r) between a specific module and RFI, with the corresponding *p*-value shown in parentheses. The color scale from blue to white to red indicates negative (blue), zero (white), and positive (red) correlations, respectively. The gray module (unassigned genes) was excluded from the analysis.

Correlation heatmaps between tissue-specific modules and RFI (Figure 4) indicated that, in the intestine, module–RFI correlation coefficients ranged from −0.21 to 0.54. Only the pink module met the significance threshold (|r| > 0.4, *p* < 0.05), with a correlation coefficient of 0.54 (*p* = 4.0 × 10^−5^). In the hepatopancreas, module–RFI correlations ranged from −0.48 to 0.44, and three modules satisfied the criteria: white (r = −0.48, *p* = 5.0 × 10^−4^), dark green (r = 0.42, *p* = 0.003), and light green (r = 0.44, *p* = 0.002). In contrast, no modules in the muscle exhibited |r| > 0.4 with *p* < 0.05. In summary, four RFI-specific modules were identified across tissues.

### 3.5. Screening of Hub Genes

**Figure 5:**
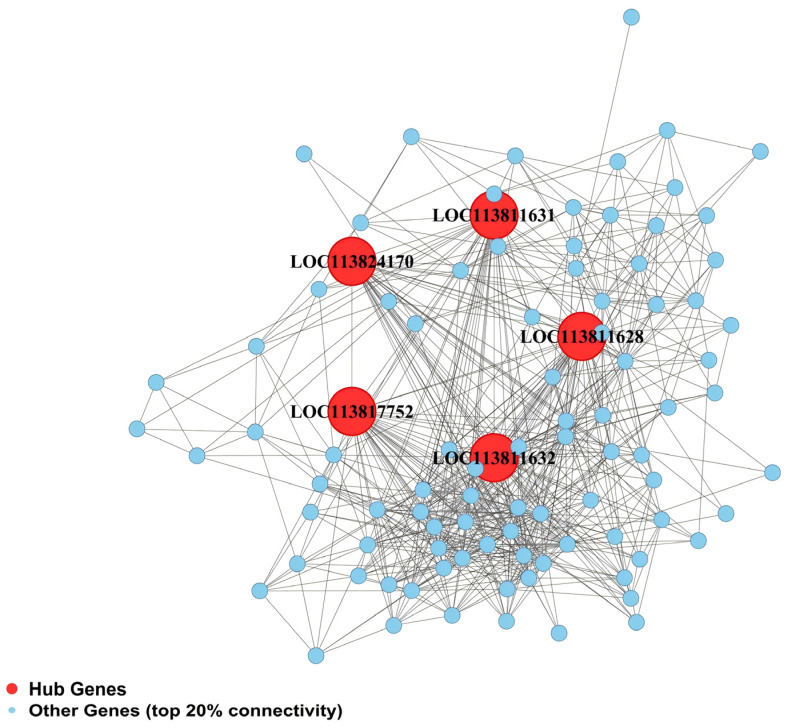
Co-expression regulatory network of hub genes in the intestine pink module.

**Figure 6:**
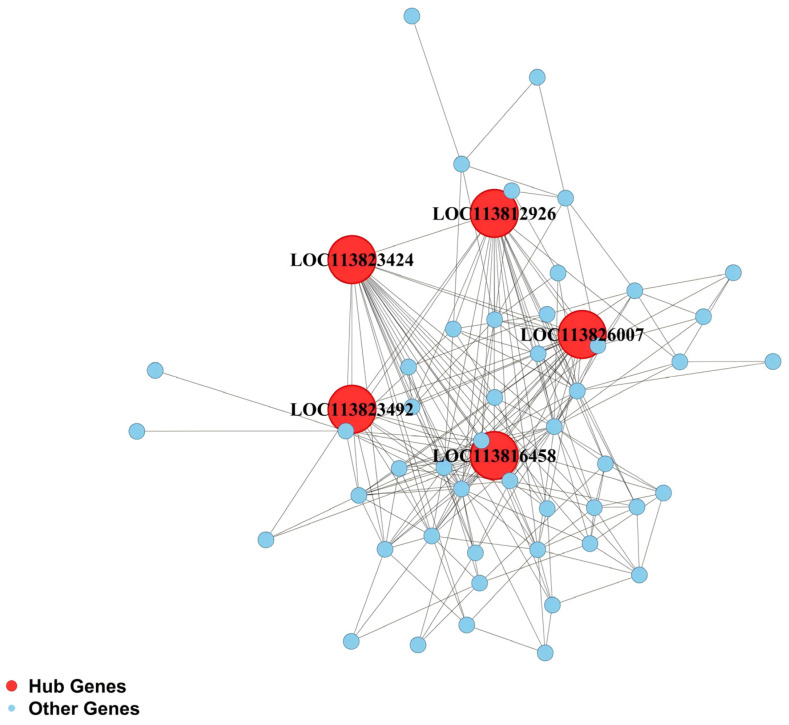
Co-expression regulatory network of hub genes in the hepatopancreas white module.

**Figure 7:**
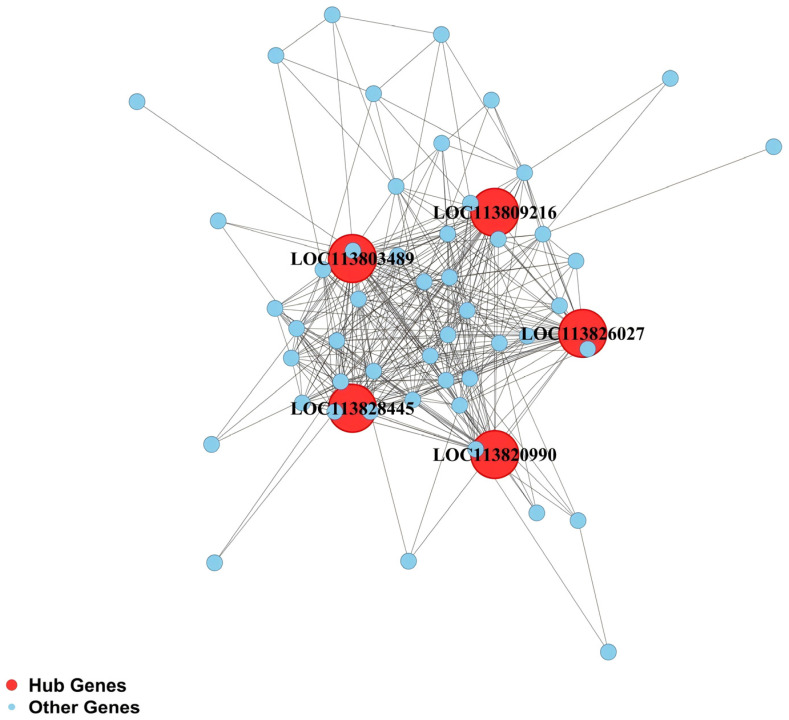
Co-expression regulatory network of hub genes in the hepatopancreas dark green module.

**Figure 8:**
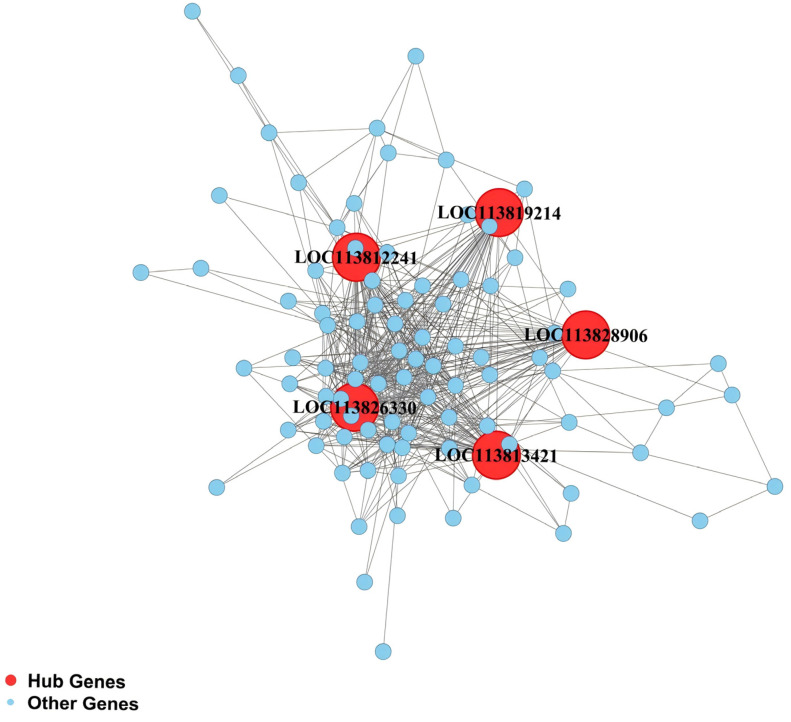
Co-expression regulatory network of hub genes in the hepatopancreas light green module.

Gene connectivity analysis was performed for the four RFI-specific modules to construct hub gene co-expression networks, and the top five hub genes per module were identified (Figure 5, Figure 6, Figure 7 and Figure 8), yielding 20 functionally annotated hub genes (Table 2).

In the intestine pink module, five hub genes were identified, primarily involved in cellular energy metabolism and mitochondrial function. *LOC113824170* and *LOC113811631* encode mitochondrial inner membrane translocase subunits Tim13 and TIM50-C, respectively, coordinating mitochondrial protein import. *LOC113811632* encodes ketohexokinase, which participates in sugar metabolism. *LOC113811628* encodes a 2-oxoglutarate and iron-dependent oxygenase domain-containing protein 3, potentially involved in redox regulation. *LOC113817752* encodes a NAP domain-containing SET protein, generally involved in chromatin modification and gene expression regulation.

In the hepatopancreas, hub genes in the white module are implicated in mitochondrial function, transcriptional regulation, and signal transduction. *LOC113826007* encodes replication in mitochondria 2, which regulates mitochondrial DNA replication. *LOC113812926* and *LOC113823492* encode zinc finger protein 836 and BTB/POZ domain-containing protein 6, respectively, likely functioning as transcriptional regulators. *LOC113823424* encodes MAP kinase-interacting serine/threonine-protein kinase 1, which is involved in MAPK pathway regulation. *LOC113816458* encodes protein fem-1 homolog C, which may participate in cell differentiation and signal transduction.

Hub genes in the dark green module are highly enriched in the V-type proton ATPase (V-ATPase) complex. These include *LOC113826027* (21 kDa proteolipid subunit c′′), *LOC113809216* (subunit D), *LOC113803489* (subunit VhaSFD), *LOC113828445* (subunit Vha44), and *LOC113820990* (ribonuclease kappa-B). The first four genes encode components of the core V-ATPase complex, which maintains intracellular pH homeostasis, lysosomal function, and vesicle transport.

Hub genes in the light green module are broadly involved in lipid metabolism and protein modification. These include *LOC113828906* (sphingomyelin phosphodiesterase), *LOC113819214* (neutral ceramidase), *LOC113812241* (pantetheinase), *LOC113826330* (astacin, a metalloprotease), and *LOC113813421* (palmitoyl-protein thioesterase 1). These genes are collectively involved in the regulation of lipid turnover, sphingolipid metabolism, and protein deacylation processes.

**Table 2:** Hub genes in RFI-specific modules.

Tissue	Module	Hub Gene	Connectivity	Description
Intestine	pink	*LOC113824170*	62.1	mitochondrial import inner membrane translocase subunit Tim13
		*LOC113811632*	61.6	ketohexokinase
		*LOC113811631*	60.6	mitochondrial import inner membrane translocase subunit TIM50-C
		*LOC113811628*	60.3	2-oxoglutarate and iron-dependent oxygenase domain-containing protein 3
		*LOC113817752*	60.1	NAP domain-containing protein SET
hepatopancreas	white	*LOC113826007*	3.566	replication in mitochondria 2
		*LOC113812926*	2.745	zinc finger protein 836
		*LOC113823424*	2.445	MAP kinase-interacting serine/threonine-protein kinase 1
		*LOC113823492*	2.370	BTB/POZ domain-containing protein 6
		*LOC113816458*	2.294	protein fem-1 homolog C
	dark green	*LOC113826027*	14.218	V-type proton ATPase 21 kDa proteolipid subunit c′′
		*LOC113809216*	13.561	V-type proton ATPase subunit D
		*LOC113803489*	13.404	V-type proton ATPase subunit VhaSFD
		*LOC113828445*	12.405	V-type proton ATPase subunit Vha44
		*LOC113820990*	11.617	ribonuclease kappa-B
	light green	*LOC113828906*	5.321	sphingomyelin phosphodiesterase
		*LOC113819214*	4.611	neutral ceramidase
		*LOC113812241*	4.286	pantetheinase
		*LOC113826330*	3.848	astacin
		*LOC113813421*	3.308	Palmitoyl-protein thioesterase 1

### 3.6. Hub Gene Expression Pattern

In the validation population, RFI in the high feed efficiency group ranged from −0.089 to −0.004 g/d (mean: −0.017 g/d), while that in the low feed efficiency group ranged from 0.006 to 0.034 g/d (mean: 0.017 g/d), showing a highly significant difference between the two groups (*p* < 0.001). Comparison of hub gene expression between groups revealed that most genes were upregulated in the high feed efficiency group (Table 3), with seven genes exhibiting *q*-values < 0.1. Notably, all intestinal candidate genes were validated, whereas only *LOC113809216* and *LOC113820990* among the hepatopancreas genes met this criterion. Furthermore, no significant correlations between gene expression and ADG were detected for six of the seven genes (*p* > 0.05, see Table 4), with the exception of *LOC113811632* (r = 0.350, *p* = 0.006).

## 4. Discussion

In this study, seven hub genes closely associated with feed utilization efficiency in *P. vannamei* fed a plant-based diet were identified through WGCNA and validated in an independent population. Among these, five genes originated from the intestine pink module (*LOC113811628*, *LOC113811631*, *LOC113824170*, *LOC113817752*, *LOC113811632*), and two genes were derived from the hepatopancreas dark green module (*LOC113809216*, *LOC113820990*). Notably, only one intestinal gene (*LOC113811632*) showed a significant correlation with ADG. The absence of significant correlations with ADG for the remaining six genes (*p* > 0.05) suggests that most of these genes are specifically related to feed utilization efficiency under plant-based diets rather than to growth regulation. In contrast, no co-expression module significantly associated with RFI was identified in the muscle tissue (|r| < 0.4). As an effector tissue primarily involved in locomotion rather than metabolic regulation, muscle may be less responsive to changes in feed utilization efficiency compared with metabolically active tissues such as the intestine and hepatopancreas. Additionally, the magnitude of gene expression differences in muscle may be relatively subtle, falling below the detection threshold set in this study. Moreover, transcriptomic variation in muscle may be predominantly driven by other factors (e.g., physical activity, individual body size) rather than directly reflecting differences in feed efficiency. The possibility of limited statistical power cannot be completely excluded, with a sample size of 50 individuals per tissue in this study. The identification of trait-specific modules and hub genes in the intestine and hepatopancreas provides valuable insights into the molecular mechanisms underlying shrimp adaptation to plant-based diets, including mitochondrial function, carbohydrate metabolism, ion transport, and nucleic acid metabolism. The functional mechanisms and regulatory logic of these hub genes are discussed below.

### 4.1. Mitochondrial Function and Protein Import

*LOC113824170* and *LOC113811631* encode the mitochondrial inner membrane translocase subunits Tim13 and TIM50-C, respectively. Both are core components of the TIM complex, which is essential for importing nuclear-encoded proteins into the mitochondrial inner membrane. The integrity and functional efficiency of the TIM complex directly determine mitochondrial protein import rates, thereby influencing overall mitochondrial function [21]. As the cellular hub of energy metabolism, mitochondrial stability and oxidative phosphorylation efficiency are crucial for maintaining cellular energy status [22]. In the high feed efficiency group, these genes were significantly upregulated in the intestine (log_2_FC = −0.343 and −0.508, *q* < 0.1). This suggests that intestinal cells may enhance mitochondrial protein import capacity to optimize mitochondrial structure and function, thereby providing sufficient energy to support the digestion and absorption of plant-based nutrients. Studies in yeast have confirmed that the TIM23 complex serves as the main translocase for matrix proteins [23], and proteomic evidence indicates that TIM complex subunit expression positively correlates with mitochondrial respiratory chain assembly efficiency [24]. Collectively, upregulation of TIM complex-related genes may enhance mitochondrial protein import capacity in the intestine, potentially supporting the increased energy demands associated with digesting and absorbing plant-based nutrients. This suggests that these genes may play a role in the adaptive response of *P. vannamei* to plant-based diets.

### 4.2. Carbohydrate Metabolism and Energy Supply

*LOC113811632* encodes ketohexokinase, a rate-limiting enzyme in fructose metabolism that directly influences cellular fructose utilization and energy supply [25]. Plant-based diets contain complex carbohydrates that must be catabolized to generate energy; therefore, enhanced activity of carbohydrate metabolism enzymes may be beneficial for energy production. In the present study, *LOC113811632* was significantly upregulated in the high feed efficiency group (log_2_FC = −0.384, *q* < 0.1), suggesting a potential role in carbohydrate utilization. This interpretation is supported by previous findings that upregulation of carbohydrate metabolism-related genes improves the utilization of plant-based diets in shrimp [9]. Moreover, studies in *P. vannamei* have shown that the regulation of glycolytic enzymes, including hexokinase, plays a critical role in metabolic adaptation to varying nutritional and environmental conditions [26]. Collectively, these lines of evidence suggest that upregulation of ketohexokinase may enhance intestinal catabolism of carbohydrates from plant-based diets, thereby contributing to energy supply and supporting nutrient absorption in *P. vannamei*.

*LOC113811628* encodes 2-oxoglutarate and iron-dependent oxygenase domain-containing protein 3, which was significantly upregulated in the high feed efficiency group (log_2_FC = −0.624, *q* < 0.1). This protein family participates in cellular redox regulation and amino acid metabolism, relying on 2-oxoglutarate and iron as cofactors [27]. Plant-based diets often contain anti-nutritional factors that can induce oxidative stress in the intestine. Upregulation of *LOC113811628* may help maintain redox homeostasis in intestinal cells, mitigating oxidative stress and thereby improving nutrient metabolic efficiency. In crustaceans, adaptive responses to dietary and environmental changes are closely linked to the regulation of metabolic enzymes [28,29], supporting the potential role of this gene in facilitating adaptation to plant-based diets.

### 4.3. Epigenetic Regulation of Metabolic Adaptation

*LOC113817752* encodes a SET protein containing a NAP domain, a member of the histone methyltransferase family. SET proteins catalyze methylation of specific lysine residues on histone H3, altering chromatin structure and regulating gene transcription, thereby participating in metabolic regulation and responses to environmental changes [30]. In the high feed efficiency group, *LOC113817752* was significantly upregulated (log_2_FC = −0.279, *q* < 0.1). Notably, its expression pattern was coordinated with that of mitochondrial metabolism-related genes, raising the possibility that this SET protein may be involved in epigenetic regulation of genes relevant to mitochondrial function. Although direct targets of this SET protein remain to be identified, and its functional role in feed efficiency requires further validation, its upregulation in high-efficiency individuals is consistent with a potential role in modulating metabolic gene expression in response to plant-based diets.

### 4.4. V-ATPase Function in Ion Transport and Lysosomal Digestion

*LOC113809216* encodes the D subunit of V-ATPase, a proton pump widely present in eukaryotic endomembrane systems. V-ATPase establishes transmembrane proton gradients via ATP hydrolysis, driving ion and nutrient transport [31]. In arthropods, V-ATPase can serve as a central energy and ion transporter, particularly in intestinal epithelia [32]. Notably, *LOC113809216* is located within the hepatopancreatic dark green module, which is also enriched with other core V-ATPase subunits, suggesting coordinated regulation of intracellular pH homeostasis and lysosomal function. Under plant-based diets, shrimp require enhanced digestion and absorption of dietary proteins. V-ATPase acidifies lysosomal compartments, increasing protease activity and facilitating the hydrolysis of dietary proteins, while also participating in membrane fusion and metabolic sensing [33]. In this study, *LOC113809216* was significantly upregulated in the high feed efficiency group (log_2_FC = −0.232, *q* < 0.1), supporting its key role in nutrient metabolism adaptation.

*LOC113820990* encodes ribonuclease kappa-B, which was significantly upregulated in the high feed efficiency group (log_2_FC = −0.288, *q* < 0.1). This gene co-localizes with core V-ATPase subunits in the hepatopancreatic dark green module, suggesting a potential functional interaction. V-ATPase-generated proton gradients are essential for lysosomal digestion and secondary active transport of nutrients [34]. Studies in insects have shown that disruption of V-ATPase subunits impairs survival, development, and gut function [35,36]. Therefore, *LOC113820990* may cooperate with V-ATPase to regulate autophagy and hepatopancreatic physiology, ultimately influencing nutrient metabolism and feed efficiency in *P. vannamei*.

### 4.5. The Application Prospects of Candidate Genes

The seven hub genes identified in this study serve as candidate targets for marker-assisted selection (MAS) for feed utilization efficiency in *P. vannamei* fed a *plant-based diet*. These genes were validated in an independent population and exhibit tissue-specific expression patterns (intestine and hepatopancreas), making them suitable for development as functional molecular markers in breeding programs. For practical implementation, SNP sites within or linked to these genes could be prioritized for developing genotyping assays to assist in the evaluation of feed efficiency in candidate individuals fed plant-based diets during early selection. It should be noted that further validation in larger populations and across different genetic backgrounds is required before commercial application. Additionally, future studies incorporating a fishmeal-based control are warranted to determine whether these genes specifically mediate adaptation to plant-protein diets or reflect general feed efficiency mechanisms. Overall, this study provides valuable molecular resources for breeding shrimp lines with improved feed efficiency under plant-based feeding regimes.

## 5. Conclusions

Through WGCNA, this study identified seven hub genes significantly associated with RFI in *P. vannamei* under a plant-based (fishmeal-free) diet, with five in the intestine (*LOC113811628*, *LOC113811631*, *LOC113824170*, *LOC113817752*, *LOC113811632*) and two in the hepatopancreas (*LOC113809216*, *LOC113820990*). Functional annotation indicates that these genes are involved in processes including mitochondrial protein transport, fructose metabolism, redox homeostasis, epigenetic modification, proton pump function, and nucleic acid metabolism. Their coordinated expression suggests a potential intestine–hepatopancreas molecular network that may contribute to enhanced energy supply, nutrient transport, and cellular homeostasis under plant-based feeding conditions. These findings provide candidate genes and mechanistic insights that may inform the development of plant-protein-based feeds and support genetic improvement strategies for feed efficiency in shrimp.

## Figures and Tables

**Table 1:** Composition of the fishmeal-free diet.

Ingredient	Proportion (g/kg)
Yeast	50
Peanut meal	170
Soybean meal	375
Wheat gluten	229.5
Soy phospholipid	15
Choline	5
Fish oil	30
Shrimp shell meal	100
Ca(H_2_PO_4_)_2_	15
Vc	0.5
Vitamin premix ^a^	5
Mineral premix ^b^	5
Proximate composition (%)	
Crude protein	38
Crude lipid	7.5
Crude fiber	3.3
Ash	6.6

^a^ Vitamin premix (mg or g/kg diet): thiamin, 25 mg; riboflavin, 45 mg; pyridoxine HCl, 20 mg; vitamin B_12_, 0.1 mg; vitamin K_3_, 10 mg; inositol, 800 mg; pantothenic acid, 60 mg; niacin, 200 mg; folic acid, 20 mg; biotin, 1.2 mg; retinol acetate, 32 mg; cholecalciferol, 5 mg; alpha-tocopherol, 120 mg; wheat middling, 3.67 g. ^b^ Mineral premix (mg or g/kg diet): MgSO_4_·7H_2_O, 1200 mg; CuSO_4_·5H_2_O, 10 mg; ZnSO_4_·H_2_O, 50 mg; FeSO_4_·H_2_O, 80 mg; MnSO_4_·H_2_O, 45 mg; CoCl_2_·6H_2_O (1%), 50 mg; NaSeSO_3_·5H_2_O (1%), 20 mg; Ca(IO_3_)_2_·6H_2_O (1%), 60 mg; zeolite, 3.485 g.

**Table 3:** Comparison of hub gene expression between the low and high feed efficiency groups.

Tissue	Module	Hub Gene	High Efficiency	Low Efficiency	log_2_FC	*p*-Value	*q*-Value
Intestine	pink	*LOC113824170*	51.565	40.647	−0.343	0.018	0.089
		*LOC113811632*	29.023	22.238	−0.384	0.027	0.091
		*LOC113811631*	16.338	11.489	−0.508	0.002	0.018
		*LOC113811628*	20.105	13.041	−0.624	0.000	0.007
		*LOC113817752*	75.260	62.011	−0.279	0.019	0.089
hepatopancreas	dark green	*LOC113809216*	389.441	331.609	−0.232	0.022	0.089
		*LOC113820990*	382.343	313.175	−0.288	0.034	0.097
		*LOC113803489*	181.113	164.899	−0.135	0.111	0.222
		*LOC113828445*	224.455	201.614	−0.155	0.095	0.212
		*LOC113826027*	462.543	431.229	−0.101	0.264	0.353
	white	*LOC113826007*	27.325	23.598	−0.212	0.184	0.287
		*LOC113812926*	28.091	24.969	−0.170	0.187	0.287
		*LOC113823424*	192.442	166.761	−0.207	0.368	0.430
		*LOC113823492*	11.326	13.288	0.230	0.221	0.316
		*LOC113816458*	31.138	24.468	−0.348	0.318	0.398
	light green	*LOC113828906*	120.259	93.283	−0.366	0.049	0.123
		*LOC113819214*	134.434	116.268	−0.209	0.171	0.287
		*LOC113812241*	201.062	182.631	−0.139	0.402	0.430
		*LOC113826330*	2424.827	2692.666	0.151	0.408	0.430
		*LOC113813421*	559.060	524.247	−0.093	0.672	0.672

Note: log_2_FC represents the log_2_ fold change of the low feed efficiency group relative to the high feed efficiency group. Negative log_2_FC indicates higher expression in the high-feed-efficiency group.

**Table 4:** Correlation between hub gene expression and ADG in all 60 individuals from both the high and low RFI groups.

Tissue	Module	Hub Gene	Correlation	*p*-Value
Intestine	pink	*LOC113811632*	0.350	0.006
		*LOC113824170*	0.082	0.535
		*LOC113811631*	0.077	0.559
		*LOC113811628*	0.125	0.340
		*LOC113817752*	0.034	0.798
hepatopancreas	dark green	*LOC113809216*	0.018	0.891
		*LOC113820990*	0.023	0.861

## Data Availability

The original contributions presented in the study are included in the article; further inquiries can be directed to the corresponding author.

## References

[1] Niu J., Zhao W. (2022). Research advances in nutritional physiology and high-efficiency and environment-friendly feed of *Litopenaeus vannamei*. J. Fish. China.

[2] Sookying D., Davis D.A. (2011). Pond production of Pacific white shrimp (*Litopenaeus vannamei*) fed high levels of soybean meal in various combinations. Aquaculture.

[3] Tacon A.G.J., Metian M. (2008). Global overview on the use of fish meal and fish oil in industrially compounded aquafeeds: Trends and future prospects. Aquaculture.

[4] Ji H., Li S.L., Xu X.X. (2016). Research progress on the application of insects as feed resources in aquaculture feed. Feed Ind..

[5] Chen F., Ding Z.R., Su Z.L., Guan J.F., Xu C., Wang S.Q., Li Y.Y., Xie D.Z. (2024). Efficiently substituting dietary fish meal with terrestrial compound protein enhances growth, health, and protein synthesis in largemouth bass. Animals.

[6] Lim C., Dominy W. (1990). Evaluation of soybean meal as a replacement for marine animal protein in diets for shrimp (*Penaeus vannamei*). Aquaculture.

[7] Dai P., Zhu X.Y., Luan S., Sui J., Meng X.H., Cao J.W., Tan J., Kong J. (2024). Quantitative genetics of feed-efficiency-related traits for the pacific whiteleg shrimp *Penaeus vannamei* in a plant-based diet environment. Biology.

[8] Liu T., Zhang G.G., Feng Y., Kong C., Ayisi C.L., Huang X.X., Hua X.M. (2019). Dietary soybean antigen impairs growth and health through stress-induced non-specific immune responses in Pacific white shrimp, *Litopenaeus vannamei*. Fish Shellfish Immunol..

[9] Zhou B., Ran H.M., Zhang Q.J., Chen H., Han F.L., Xu C., Zhao Q. (2024). Unveiling the impact of rapeseed meal on feeding behavior and anorexigenic endocrine in *Litopenaeus vannamei*. Animals.

[10] Widmann P., Reverter A., Weikard R., Suhre K., Hammon H.M., Albrecht E., Kuehn C. (2015). Systems biology analysis merging phenotype, metabolomic and genomic data identifies *Non-SMC Condensin I Complex, Subunit G* (*NCAPG*) and cellular maintenance processes as major contributors to genetic variability in bovine feed efficiency. PLoS ONE.

[11] Tian Z.L., He W.X., Tang J.N., Liao X., Yang Q., Wu Y.M., Wu G.S. (2020). Identification of important modules and biomarkers in breast cancer based on WGCNA. Onco Targets Ther..

[12] Wang X.P., Liu H.L., Zhang D., Zou D.T., Wang J.G., Zheng H.L., Jia Y., Qu Z.J., Sun B., Zhao H.W. (2022). Photosynthetic carbon fixation and sucrose metabolism supplemented by weighted gene co-expression network analysis in response to water stress in rice with overlapping growth stages. Front. Plant Sci..

[13] Fu Z.Q., Lin Z.Y., Huang K.Q., Li Z.F., Luo Z., Han F.L., Li E.C. (2024). Dinotefuran exposure alters biochemical, metabolomic, gut microbiome, and growth responses in decapoda pacific white shrimp *Penaeus vannamei*. J. Hazard. Mater..

[14] Fan Y., Cao L., Liang Y., Feng B., Gong J., Li J., Chen H., Zhang M., Feng J. (2025). Transcriptomic WGCNA analyses reveal sectional regulatory mechanisms in giant freshwater prawn *Macrobrachium rosenbergii* larvae under hypoxia-reoxygenation. Comp. Biochem. Physiol. Part D Genom. Proteom..

[15] Jahan K., Yin Z., Zhang Y., Yan X., Nie H. (2022). Gene co-expression network analysis reveals the correlation patterns among genes in different temperature stress adaptation of Manila Clam. Mar. Biotechnol..

[16] Xu H.G., Zhang Y.L., Luo K., Meng X.H., Luan S., Cao B.X., Chen B.L., Liang M.Q., Kong J. (2017). Arachidonic acid in diets for early maturation stages enhances the final reproductive performances of Pacific white shrimp (*Litopenaeus vannamei*). Aquaculture.

[17] Chen S.F., Zhou Y.Q., Chen Y., Gu J. (2018). Fastp: An ultra-fast all-in-one FASTQ preprocessor. Bioinformatics.

[18] Yuan J.B., Zhang X.J., Wang M., Sun Y.M., Liu C.Z., Li S.H., Yu Y., Gao Y., Liu F., Zhang X.X. (2021). Simple sequence repeats drive genome plasticity and promote adaptive evolution in penaeid shrimp. Commun. Biol..

[19] Kim D., Langmead B., Salzberg S.L. (2015). HISAT: A fast spliced aligner with low memory requirements. Nat. Methods.

[20] Anders S., Pyl P.T., Huber W. (2015). HTSeq--a Python framework to work with high-throughput sequencing data. Bioinformatics.

[21] Chacinska A., Koehler C.M., Milenkovic D., Lithgow T., Pfanner N. (2009). Importing mitochondrial proteins: Machineries and mechanisms. Cell.

[22] Morelli A.M., Scholkmann F. (2024). Should the standard model of cellular energy metabolism be reconsidered? Possible coupling between the pentose phosphate pathway, glycolysis and extra-mitochondrial oxidative phosphorylation. Biochimie.

[23] Demishtein-Zohary K., Azem A. (2017). The TIM23 mitochondrial protein import complex: Function and dysfunction. Cell Tissue Res..

[24] Schmidt O., Pfanner N., Meisinger C. (2010). Mitochondrial protein import: From proteomics to functional mechanisms. Nat. Rev. Mol. Cell Biol..

[25] Tee S.S., Kim N., Cullen Q., Eskandari R., Mamakhanyan A., Srouji R.M., Chirayil R., Jeong S., Shakiba M., Kastenhuber E.R. (2022). Ketohexokinase-mediated fructose metabolism is lost in hepatocellular carcinoma and can be leveraged for metabolic imaging. Sci. Adv..

[26] Flores-Sauceda M.A., Leyva-Carrillo L., Camacho-Jiménez L., Gómez-Jiménez S., Peregrino-Uriarte A.B., Yepiz-Plascencia G. (2024). Two hexokinases of the shrimp *Penaeus (Litopenaeus) vannamei* are differentially expressed during oxygen limited conditions. Comp. Biochem. Physiol. A Mol. Integr. Physiol..

[27] Islam M.S., Leissing T.M., Chowdhury R., Hopkinson R.J., Schofield C.J. (2018). 2-Oxoglutarate-Dependent Oxygenases. Annu. Rev. Biochem..

[28] Zhang X.X., Zhu H.C., Yuan J.B., Zhang X.J., Xiang J.H., Li F.H. (2024). Diversity of heat shock proteins in response to various stressors in the Pacific white shrimp *Litopenaeus vannamei*. Aquaculture.

[29] Zhan Q.Y., Han T., Li X.Y., Wang J.T., Yang Y.X.J., Zheng P.Q., Liu T., Xu H.Y.L., Wang C.L. (2020). Effects of dietary carbohydrate levels on growth, body composition, and gene expression of key enzymes involved in hepatopancreas metabolism in mud crab *Scylla paramamosain*. Aquaculture.

[30] Dillon S.C., Zhang X., Trievel R.C., Cheng X.D. (2005). The SET-domain protein superfamily: Protein lysine methyltransferases. Genome Biol..

[31] Forgac M. (2007). Vacuolar ATPases: Rotary proton pumps in physiology and pathophysiology. Nat. Rev. Mol. Cell Biol..

[32] Klein U. (1992). The insect V-ATPase, a plasma membrane proton pump energizing secondary active transport: Immunological evidence for the occurrence of a V-ATPase in insect ion-transporting epithelia. J. Exp. Biol..

[33] Maxson M.E., Grinstein S. (2014). The vacuolar-type H^+^-ATPase at a glance—More than a proton pump. J. Cell Sci..

[34] Shi X.K., Liu X.J., Cooper A.M.W., Silver K., Merzendorfer H., Zhu K.Y., Zhang J.Z. (2022). Vacuolar (H^+^)-ATPase subunit c is essential for the survival and systemic RNA interference response in *Locusta migratoria*. Pest. Manag. Sci..

[35] Rahmani S., Bandani A.R. (2021). A gene silencing of V-ATPase subunit A interferes with survival and development of the tomato leafminer, *Tuta absoluta*. Arch. Insect Biochem. Physiol..

[36] Li Y., Ze L.J., Liu F.J., Liao W., Lu M., Liu X.L. (2022). RNA interference of vATPase subunits A and E affects survival of larvae and adults in *Plagiodera versicolora* (*Coleoptera: Chrysomelidae*). Pestic. Biochem. Physiol..

